# Neglecting diurnal variations leads to uncertainties in terrestrial nitrous oxide emissions

**DOI:** 10.1038/srep25739

**Published:** 2016-05-09

**Authors:** Narasinha J. Shurpali, Üllar Rannik, Simo Jokinen, Saara Lind, Christina Biasi, Ivan Mammarella, Olli Peltola, Mari Pihlatie, Niina Hyvönen, Mari Räty, Sami Haapanala, Mark Zahniser, Perttu Virkajärvi, Timo Vesala, Pertti J. Martikainen

**Affiliations:** 1 Biogeochemistry research group, Department of Environmental and Biological Sciences, University of Eastern Finland, Yliopistoranta 1D E, PO Box 1627, Kuopio campus, FI-70211 Finland; 2 Department of Physics, P.O. Box 48, University of Helsinki, 00014 Finland; 3 Department of Food and Environmental Sciences, Division of Microbiology and Biotechnology, P.O. Box 56, University of Helsinki, FI-00014 Finland; 4 Natural Resources Institute Finland, Green technology, Halolantie 31A Maaninka FI-71750, Finland; 5 Aerodyne Research, Inc., 45 Manning Road Billerica, MA 01821-3976, USA; 6 Viikki Plant Science Centre, University of Helsinki, P.O. Box 27, FI-00014 Finland; 7 Department of Forest Sciences, P. O. Box 27, University of Helsinki, 00014 Finland

## Abstract

Nitrous oxide (N_2_O) is an important greenhouse gas produced in soil and aquatic ecosystems. Its warming potential is 296 times higher than that of CO_2_. Most N_2_O emission measurements made so far are limited in temporal and spatial resolution causing uncertainties in the global N_2_O budget. Recent advances in laser spectroscopic techniques provide an excellent tool for area-integrated, direct and continuous field measurements of N_2_O fluxes using the eddy covariance method. By employing this technique on an agricultural site with four laser-based analysers, we show here that N_2_O exchange exhibits contrasting diurnal behaviour depending upon soil nitrogen availability. When soil N was high due to fertilizer application, N_2_O emissions were higher during daytime than during the night. However, when soil N became limited, emissions were higher during the night than during the day. These reverse diurnal patterns supported by isotopic analyses may indicate a dominant role of plants on microbial processes associated with N_2_O exchange. This study highlights the potential of new technologies in improving estimates of global N_2_O sources.

Nitrous oxide (N_2_O) is among the most important greenhouse gases (GHGs). It is produced both in natural and managed soils, with agricultural soils being the largest anthropogenic source^1^,^2^. Despite the recent progress in quantifying the diverse N_2_O sources, the range of the global estimate is still large varying from 8.1–30.7 Tg N (N_2_O) a^−1^ (across all natural and anthropogenic sources) owing primarily to the staggering spatiotemporal variation in the fluxes^3^. Recently, attempts have been made to present a comprehensive estimate of the global N_2_O emissions using observations, bottom-up and inversion models^4^. Despite the utilization of all currently available global data on N_2_O emissions from various source sectors, the overarching goal of reducing the large uncertainty in global N_2_O budget still remains a formidable task. Additionally, our current process level understanding of the factors regulating N_2_O emissions at a given site is far from complete^5^,^6^.

As a general method, static chambers are widely used to measure soil N_2_O fluxes experimentally^7^,^8^,^9^,^10^,^11^. Most N_2_O exchange measurements are made using manual chambers leading to sparse temporal resolution in the data. To overcome these discrepancies, micrometeorological methods (such as the eddy covariance (EC) technique) are being increasingly applied for continuous measurements of N_2_O exchange^12^,^13^,^14^. This method offers the advantage of integrating N_2_O fluxes over larger areas. Until recently, however, the lack of appropriate gas analysers with a sufficient time response and precision has been a major limitation. With the recent development of laser-based spectroscopic techniques, fast and accurate measurements in conjunction with the EC or automatic chamber techniques are now possible^14^,^15^,^16^,^17^,^18^ allowing accurate studies on the temporal variation of N_2_O fluxes.

As a part of the Integrated Carbon Observation System (ICOS), a European infrastructure program dedicated to high precision monitoring of greenhouse gases, we conducted an EC based inter-comparison field campaign^18^ utilizing four different N_2_O analyzers. Nitrous oxide flux measurements were made over an agricultural field site (in eastern Finland) cultivated with reed canary grass (RCG, *Phalaris arundinaceae*, L.), a perennial crop, from April until November in 2011 (Supplementary Information).

The soil moisture and temperature during the period (Supplementary Fig. S1) were typical of conditions during late spring, summer and early autumn in this ecosystem. The mean volumetric water content in the soil profile (0–100 cm deep) during early May was high (0.63 ± 0.1 cm^3^ cm^−3^) owing to the melting of snow since April. With soil profile temperatures steadily increasing from 2.5 °C (with a standard deviation of 2.5 °C) in early May to 11.6 °C ( ± 2.7 °C) by early June, moisture content in all soil layers showed a steady decline. Soil moisture content remained stable at 0.25 cm^3^ cm^−3^ ( ± 0.04 cm^3^ cm^−3^) during June to mid-September. The fluctuations in soil moisture content were less pronounced with soil depth. Soil temperatures peaked at 15.7 °C ( ± 1.6 °C) during the second week of July. Towards the end of the study period, soil moisture content was high and soil temperatures were low. The O_2_ concentrations in the first 30 cm of the soil profile stayed high (between 15 and 21%) throughout the observation period (Supplementary Fig. S1). However, O_2_ concentrations were low and varied widely at deeper soil layers indicating oxygen limitations in these layers. Water filled pore space (WFPS) which regulates soil gas diffusion rate, varied between 60–100% during the early part, 20–60% during the middle part of the season and remained above 60% during the later part of the study period (Supplementary Fig. S1) with the surface soil layers generally exhibiting higher variation in WFPS throughout the season.

An emission pulse (up to 33 mg m^−2^ d^−1^) that occurred within 24 hours of the application of an N-P-K-S fertilizer containing 76 kg N ha^−1^ characterized the seasonal pattern of N_2_O exchange from the RCG cultivation system. This high N_2_O emission activity lasted about 15 days (days 143–158, referred to hereafter as the high emission period, Fig. 1A). Following this period, emissions declined to background levels of about 1 mg m^−2^ d^−1^ (low emission period). All operational N_2_O gas analyzers showed a similar seasonal pattern of N_2_O exchange. It is relevant here to dwell on the seasonal plant growth pattern in conjunction with the above-indicated seasonal trend in N_2_O exchange. Soil moisture levels were optimal and the daily precipitation was well distributed during the early part of the growing season (Fig. 1B). This resulted in a sharp increase in the crop growth (represented in Fig. 1C as gross ecosystem productivity, GEP) with GEP reaching its peak about three weeks following the fertilizer application and the largest N_2_O release to the atmosphere during the season.

As the EC method offers unique possibilities for insights into N_2_O fluxes at high temporal resolution^6^, we focused here particularly on diurnal variation in N_2_O flux as measured by one of the analyzers (considered to be the best performing N_2_O analyzer in an inter-comparison study)^18^. During the high flux period shown in Fig. 2A, N_2_O emissions exhibited a diurnal variation, with the highest fluxes observed during the daytime and the lowest at nighttime on three consecutive days. All analyzers operational during this time exhibited a similar pattern. The daytime emissions were 2.7–4.2 times higher than those measured during night (Fig. 2A). A mean diurnal pattern for the entire high-flux period (n = 16) is shown in Fig. 2B. The subsequent, much longer low flux period (day 161– day 273) revealed some peculiarities in the temporal N_2_O dynamics. A closer look at the diurnal pattern of N_2_O emissions during this N deficient low flux period indicated that N_2_O emissions were, on an average, about 50% higher during night than during the day (Fig. 3A). This is in contrast to the diurnal trend observed during the high-flux period (Fig. 2B). Importantly, all analyzers operational during the time were consistent in recording such a phenomenon.

The seasonal (April – November 2011) sum of N_2_O emissions amounted to 2.8 kg N_2_O ha^−1^ with emissions from the short high flux period accounting for about 55% of the seasonal sum. As discussed above, the diurnal N_2_O exchange during and outside the high flux period were in contrast. Total emissions from the high flux period calculated only from daytime measured fluxes (and thereby neglecting the diurnal variations) were 23% higher compared to the sum calculated by considering the diurnal pattern. In contrast to this overestimation, the total emissions during N deficient conditions were 16% lower compared to the sum obtained by accounting for the complete diurnal pattern of N_2_O exchange.

We carried out an independent process study; a ^15^N labelling (^15^NH_4_^15^NO_3_) field campaign during mid-July in 2011 (the low-flux period) on plots cultivated with a mixture of timothy (Phleum pratense L.) and meadow fescue (Festuca pratensis Huds.) and located at the same study site. Repeated measurements of ^15^N atom % abundance of accumulated N_2_O in the headspace of opaque and transparent chambers (simulating night and day-time conditions) were consistently and significantly different. While the ^15^N atom % values of N_2_O from transparent chambers remained about constant during the chamber closure period, those from the opaque chambers increased linearly over the 35 minutes of chamber closure (Fig. 4). The slope of the build-up of the heavier isotope (^15^N) in the dark chamber headspace was an order of magnitude higher (0.001 atom % min^−1^) compared to that in the transparent chamber headspace (0.0001 atom % min^−1^).

In terrestrial ecosystems, N_2_O is produced via a wide range of microbiological N cycling processes such as chemolithotrophic NH_4_^ + ^oxidation^19^, denitrification^20^,^21^, nitrifier denitrification^22^, codenitrification^23^, dissimilatory nitrate reduction (DNRA)^24^, heterotrophic nitrification^25^,^26^, chemodenitrification and abiotic decomposition of ammonium nitrate^27^. The factors that regulate N cycling processes of denitrification and nitrification in soils have been categorized as proximal and distal controls^28^. For denitrifying microbes the key proximal factors directly controlling the activity are O_2_ concentration, soil NO_3_^−^ and C availability and temperature^29^,^30^. For nitrification, availability of O_2_ and NH_4_^ + ^and temperature are important proximal controls^31^. Distal factors such as plant growth, management practices, soil texture and water availability, regulate indirectly denitrification/nitrification activities by affecting the proximal controlling factors^28^,^29^,^30^.

We observed the largest N_2_O emissions at the study site immediately after the application of an inorganic N fertilizer (Fig. 1A). During this high emission period with the daytime peaks in N_2_O emissions, availability of mineral N (NH_4_^ + ^and NO_3_^−^) was high (Supplementary Fig. S2). It is noteworthy at this juncture that the perennial bioenergy crop (RCG) from the previous season was left on the site to overwinter. RCG possesses a large amount of nonstructural carbohydrate (NSC) reserves in its roots as a coping mechanism to overcome the harsh overwintering environment. The concentration of NSCs in the RCG roots has been reported to be about three times higher than that in the aboveground plant parts^32^. The site was fertilized within a month after the crop from the previous season was harvested. Wetting events and the presence of RCG litter left over on the soil surface after crop harvesting as well as high activity of RCG roots may have aided the development of anaerobic microsites in the surface layers^33^. Lack of oxygen (Supporting Fig. 2), high soil SOC and high availability of soil N may have triggered enhanced rates of N_2_O production through denitrification.

Under conditions of high mineral N availability, the variation in temperature likely was among the key factor for the diurnal variation in the microbial activities associated with N_2_O emissions. Diurnal variability with a daytime peak in N_2_O fluxes was reported for the first time in the late 1970s^34^,^35^. The first study^36^ that specifically examined reasons for the diurnal variability attributed 90% of the observed diurnal variation to changes in soil temperature and associated changes in N_2_O solubility. Another study^37^ considers that the role of the plant photosynthetic activity in the N_2_O flux diurnal variability is caused by supplying C to soil microorganisms, via root exudation, since days with large diurnal variability in N_2_O fluxes had high radiation. This could lead also to lower oxygen content in the soil surface during the daytime. Such a day-night pattern in oxygen content was noticed at our study site (Supplementary Fig. 2). A study conducted in 2010^38^ observed diurnal variation in N_2_O flux during a brief period of high N_2_O flux following the cultivation of a native temperate grassland soil. Based on isotopologue data, the aforementioned study reported an increase in N_2_O derived from bacterial denitrification from morning to afternoon hours.

As indicated above, the high flux period following the fertilizer application lasted about 15 days. Thereafter, the soil mineral N status was consistently low (Supplementary Fig. S2), while the RCG crop was actively photosynthesizing (Fig. 3B). Once the effect of the applied N subsides, soils generally emit N_2_O at small rates, often described as the ‘background’ emissions. During this ‘background’ emission phase lasting until the end of the plant photosynthetic activity, a diurnal pattern with lower daytime net emissions compared to those during the nighttime was consistently evident (Fig. 3A). Such observations with low net emissions or even net soil N_2_O uptake during daytime have been reported previously^39^,^40^. However, this is one of the few studies that report such continuous records of this phenomenon with such high precision in a perennial grassland ecosystem under Nordic conditions.

A number of studies have reported a sharp increase in N_2_O concentration with depth^25^,^41^. In these studies, N_2_O concentrations have been as high as 20–30 times the ambient concentration in the atmosphere at the deepest subsoil sampling points, suggesting N_2_O production rate in subsoil is sufficient to maintain a steep N_2_O concentration gradient through the soil profile. Based on the soil moisture content, O_2_ concentration and WFPS patterns at our site, it is intuitive to assume that there is a continuous N_2_O production deeper in the soil profile throughout the growing season. However, based on the data we have, it is not clear how this deep N_2_O could contribute to the contrasting diurnal variation in the N_2_O emissions within the growing season, since the diurnal variations in all environmental variables such as soil oxygen and water content, temperature, especially at deeper layers, are small (Supplementary Fig. 2).

During active photosynthesis, plant roots discharge readily available C^42^. The root exudation process is reported to follow diurnal rhythms, with exudation increasing during light periods^43^. Such observations suggest that the transfer of C compounds from assimilating leaves to roots and soil microbes occurs fast, within hours, as has been shown for RCG by using ^13^CO_2_ pulse-chase labeling^44^, demonstrating the tight coupling of soil microbial activity to plant photosynthesis^45^. This extra carbon stimulates the activity of heterotrophic microbes and immobilization of inorganic N. Additionally, soil available N is taken up by the plants during daytime^46^. This leads to a shortage of 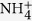 that limits the activity of ammonium oxidizing microbes and their N_2_O production. With more carbon available for the denitrifiers, enhanced rates of N_2_O reduction to N_2_ are supported under poor N availability and lower oxygen content in the soil profile owing to increased heterotrophic and autotrophic respiration^47^. Therefore, it is logical to think that the amount of N_2_O that diffuses from the deeper layers during daytime is likely to be consumed by denitrifiers more efficiently thus lowering the overall N_2_O emissions. The relatively higher reduction of N_2_O to N_2_ may help explain the negative relationship between the gross ecosystem productivity and net N_2_O emissions (Fig. 3C).

The soil oxygen concentrations in the surface layers exhibited diurnal variations in the surface layers (0–20 cm depths) during May through August (Supplementary Fig. 2). This is the period when the plants are photosynthetically most active. The variations were dampened during the month of September when the plants were undergoing senescence. Additionally, there were no consistent diurnal variations in oxygen concentrations deeper in the soil layer (80–100 cm) suggesting that these deeper layers are decoupled with respect to the plant root activity in the upper layers. Our assumption that the plants have a role to play in C and N dynamics finds additional support in the results from an independent ^15^N labelling campaign (Fig. 4), mentioned above. After adding labelled fertilizer to the soil, the label was tracked in N_2_O to a greater extent under dark than under light conditions. While the ecosystem scale EC measurements represented N_2_O emissions from RCG, a perennial grass species with aerenchymatous tissue, the plot scale isotopic analyses were performed on timothy and meadow fescue, perennial grass species with no such tissue. These common observations from this study highlighting light-dependent plant and soil microbial interactions from structurally different plant species suggest that the N dynamics perhaps follow a similar pattern under N deficiency across vegetation types in this ecosystem.

Most of the past studies on N cycling in agricultural soils have focused their attention primarily on plant available N in soluble inorganic (NO_3_^−^, NH_4_^ + ^, NO_2_^−^) form. Recently, however, the thought that plants may rely on organic N sources when the effect of the added N fertilizer subsides is gaining importance^48^. In view of this, the original hole in the pipe model with only two N sources is now suggested to be modified to include organic N as an additional N source in the pipe^49^. The pool of soluble organic N, especially free amino acids, has been observed in soils at concentrations comparable to or greater than that of inorganic N^50^. Soil organic matter turnover, plant, and microbial N uptake processes operate rapidly and at varying rates, causing soil amino acid composition to be highly dynamic in space and time^51^. A study on soil N flux dynamics in fertilized and unfertilized boreal forest soils has shown that soil amino acids can vary over short, diurnal timescales^52^. In view of such observations, it is possible that the dynamic nature of soil organic N plays a role in the diurnal regulation of N_2_O emissions from our study site, especially under conditions of low soil N availability. However, effects of environmental change on soil amino acid concentrations and composition and their role in soil N transformations are not yet well understood.

Where does the N_2_O emitted during the high and low flux periods originate from ? What are the dominant processes that account for the contrasting diurnal patterns of N_2_O emissions ? These obvious questions need further studies on soil physical, chemical and biological properties and plant-microbial processes. Our observations highlight that the accurate net N_2_O exchange across the soil–atmosphere interface, significantly improved by modern analytical methods such as the continuous wave laser spectroscopy, can now be measured to reveal contrasting short-term exchange patterns under high and low soil N availability. Such studies need to be ideally combined with smaller scale process studies on a higher spatial and temporal resolution to elucidate the soil processes and drivers of simultaneous N_2_O production, consumption and transport within the rhizosphere. In view of the dynamic short-term variations in N_2_O emissions, continuous automated chamber- and eddy covariance-based flux measurements are necessary for a better understanding of the intimate, temporal coupling between plants and soil microbial communities and their resource demands in terrestrial ecosystems.

## Methods

### The study site

The eddy covariance measurements of N_2_O exchange were made on an agricultural field site, 6.3 ha in area. The site is located in the rural district of Maaninka, Eastern Finland (63° 9′ 48.69″ N, 27° 14′ 3.29″ E). Long-term (reference period 1981–2010) annual air temperature in the region is 3.2 °C and the annual precipitation is 612 mm^53^. The soil type varied between loam and clay loam. The field was sown in June 2009 with a perennial bioenergy crop, reed canary grass (RCG, *Phalaris arundinaceae*, L. cv. Palaton). After the first year of crop establishment, the site was fertilized at the start of every growing season (late May) with an N-P-K-S fertilizer containing 76 kg N ha^−1^ (NO_3_-N : NH_4_-N = 47:53). The canopy height developed throughout the 2011 growing season from about 10 cm in mid-May to 1.7 m by late June with a slower increase to a maximum height of 1.9 m by early July (see Supplementary Information for further details about the site).

### Eddy covariance (EC) measurements of N_2_O and CO_2_ exchange

The EC measurements were made as a part of the ICOS (Integrated Carbon Observation System) Finland program during April to November 2011^18^. The main objective behind these measurements was to measure accurately the nitrous oxide exchange from an agricultural site and to compare the performance of four different laser-based commercial gas analyzers. The EC system consisted of four different N_2_O gas analyzers: ARC – continuous wave laser spectrometer, Aerodyne Research Inc., Billerica, MA, USA; TGA – trace gas analyser, Campbell Scientific Inc., Logan, UT, USA; continuous wave laser spectrometer, Los Gatos Research Inc., Mountain View, CA, USA; ARP – pulsed quantum cascade laser spectrometer, Aerodyne Research Inc., Billerica, MA, USA (see Supplementary Information for details). While TGA is a legacy trace gas analyser, ARP represents the intermediate level and ARC and LGR represent the highest levels of advancement with respect to their ability to detect N_2_O signals at a precision necessary for EC measurements. Careful calibration and corrections were performed in order to meet the objectives of the campaign. In addition, two sonic anemometers (RS-50, Gill Solent Ltd., UK and USA-1, Metek Germany GMBH) for measuring turbulent wind components, an infrared gas analyser (LI7000/LI6262 – Li-Cor Inc., Lincoln, NE, USA) for detecting high frequency CO_2_ and water vapour concentrations were used in the study. A weather station located at the site monitored continuously several micrometeorological observations such as air temperature and humidity, atmospheric pressure, wind speed and direction, radiation balance components, photosynthetically active radiation (PAR), profiles of soil temperature and moisture etc. Greater details on EC data acquisition, adopted data processing procedures, applied flux corrections and instrument details are described in the Supplementary Information.

### Isotopic analyses of ^15^N

Chamber technique based nitrous oxide fluxes were measured on 8 July 2011 from pre-installed aluminium collars (0.36 m^−2^ basal area) with transparent chamber and opaque chamber (with a chamber height of 0.33 m and 0.15 m, respectively) from 3 plots (9 m^2^ each). The plots were established in June 2009 with a forage grass mixture consisting of timothy (*Phleum pratense* L. ,cv. Tuukka), and meadow fescue (*Festuca pratensis* Huds., cv Antti). Grass field had been harvested two weeks earlier (on 22 June 2011) and a second fertilizer dose of ^15^N-enriched Ammonium Nitrate (^15^NH_4_^15^NO_3_, 10 AT-% (atom percent abundance) in both N forms at a rate of 100 kg N ha^−1^) was applied a day prior to the chamber flux measurements. The objective of this field campaign was to estimate the fraction of N_2_O derived from the ^15^N-enriched N-fertilizer.

Each N_2_O flux measurement lasted 35 min. Gas samples for AT-% of ^15^N-N_2_O analysis were collected at 5, 10, 15, 25 and 35 min intervals from the chamber headspace. Gas samples were taken with a 160 ml syringe from which the sample was immediately injected into a pre-evacuated 120 ml vials. For transparent chamber measurements, as the grass species fixed CO_2_ from the chamber head space under light, concentration inside the chamber was monitored continuously with an infrared gas analyser (LI6400, Li-Cor Inc., Lincoln, NE, USA) over the 35 min chamber deployment duration. CO_2_ levels inside the transparent chamber were continuously adjusted during this period by adding CO_2_ gas into the chamber air from a standard CO_2_ gas bottle so that the CO_2_ concentration within the chamber remained close to the ambient level over the measurement period. Additionally, a cooling unit was used in the transparent chamber measurement to keep the chamber air temperature close to the outside air temperature. During the opaque chamber measurement, however, no chamber air cooling or CO_2_ level adjustment was done. The 35 min average temperatures from transparent and dark chamber headspaces varied within 5 °C.

The collected gas samples were analysed for the AT -% ^15^N-N_2_O with an isotope ratio mass spectrometer (IRMS; Thermo Finnigan Delta V plus with ConFlo IV, Thermo Fisher Scientific) coupled to a trace-gas pre-concentration unit (PreCon, Thermo Fisher Scientific) and a gas chromatograph. Water and CO_2_ gas were removed from the sample gas by a chemical trap (containing NaOH for CO_2_ and Mg(ClO_4_)_2_ for H_2_O).

## Additional Information

**How to cite this article**: Shurpali, N. J. *et al*. Neglecting diurnal variations leads to uncertainties in terrestrial nitrous oxide emissions. *Sci. Rep*. **6**, 25739; doi: 10.1038/srep25739 (2016).

## Supplementary Material

Supplementary Information

## Figures and Tables

**Figure 1:**
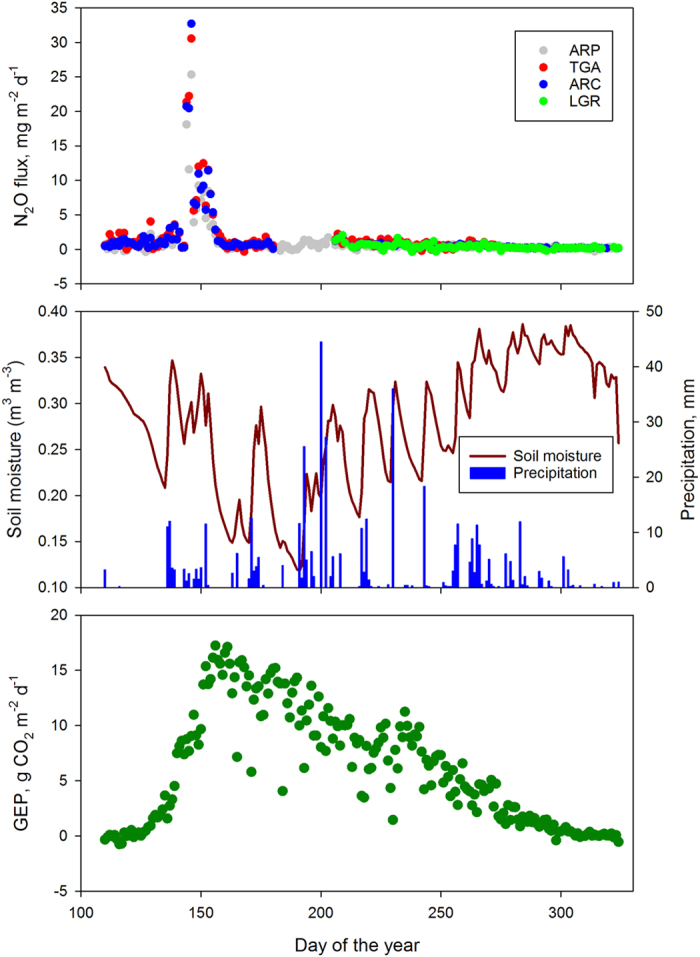
Seasonal distribution of (**A**) daily averaged N_2_O emissions (mg m^−2^ d^−1^) as measured by 4 different laser based gas analysers (**ARC** – continuous wave laser spectrometer, Aerodyne Research Inc., Billerica, MA, USA; **TGA** – trace gas analyser, Campbell Scientific Inc., Logan, UT, USA; **LGR** - continuous wave laser spectrometer, Los Gatos Research Inc., Mountain View, CA, USA; **ARP** – pulsed quantum cascade laser spectrometer, Aerodyne Research Inc., Billerica, MA, USA) (**B**) daily averaged volumetric soil moisture content (m^3^ m^−3^) measured at 2.5 cm below the soil surface and daily sum of precipitation (mm) and (**C**) daily sums of gross ecosystem productivity (GEP, g C m^−2^ d^−1^) during the N_2_O instrument inter-comparison organised during April – November 2011 at a mineral soil site at Maaninka in eastern Finland. The site was cultivated with reed canary grass (RCG, *Phalaris arundinaceae*, L.), a perennial bioenergy crop.

**Figure 2:**
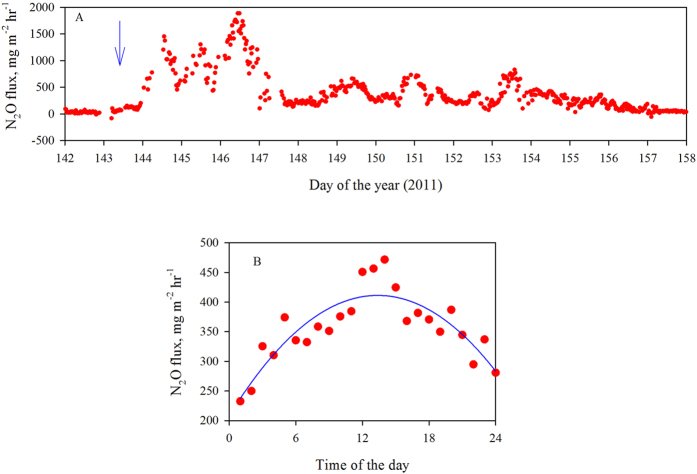
Evolution of 30-minute values of (**A**) N_2_O exchange in μg m^−2^ hr^−1^ (red solid circles, the downward arrow indicates the day of fertilization with ammonium nitrate), (**B**) mean diurnal flux pattern during the high flux period (red solid circles; the black line is a trendline fitted to the mean diurnal flux data in μg m^−2^ hr^−1^ with the quadratic least squares fit). Measurements were made at a mineral soil site located in Maaninka in eastern Finland during April – November 2011. The site was cultivated with reed canary grass (RCG, *Phalaris arundinaceae*, L.), a perennial bioenergy crop.

**Figure 3:**
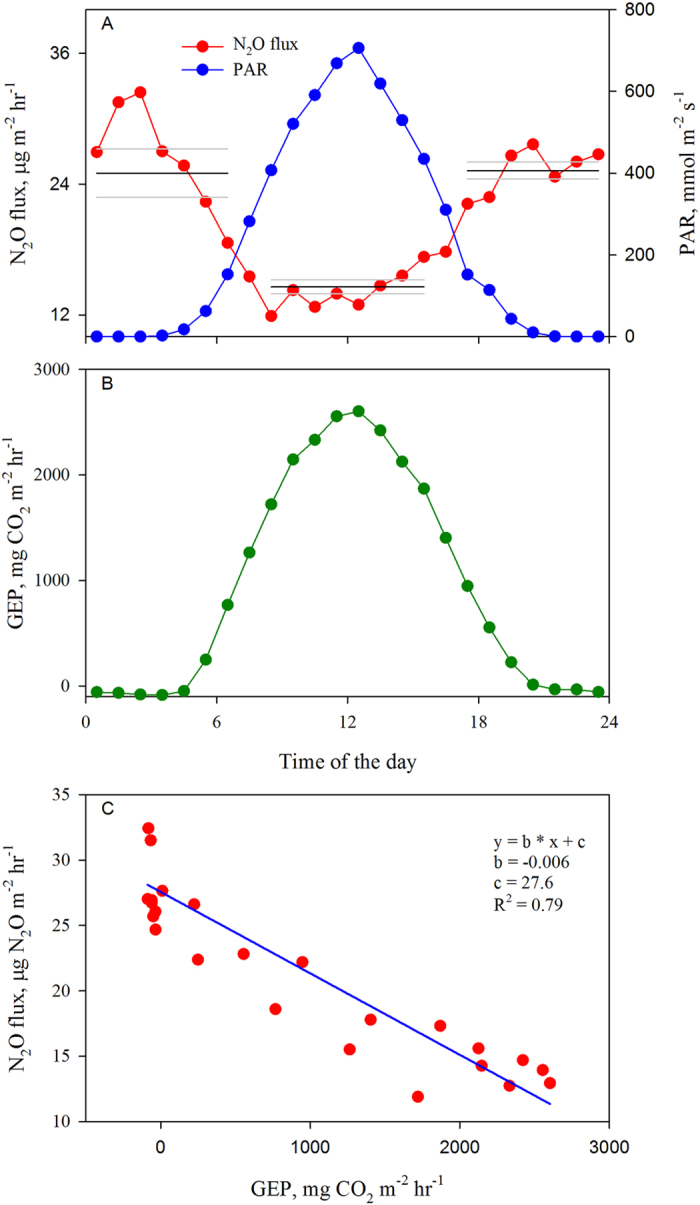
Mean (day 206– day 280) diurnal pattern of (**A**) N_2_O exchange in μg m^−2^ hr^−1^ (red solid circles) and photosynthetically active radiation (PAR in μmol m^−2^ s^−1^, blue solid circles where solid black lines represent average N_2_O emission rates during different periods of the day, light grey lines above and below the black line represent the confidence band with ± 1 standard error of the mean), (**B**) gross ecosystem productivity (GEP, mg CO_2_ m^−2^ hr^−1^, green solid circles) during the low flux period (day 206–280) in 2011 growing season at a mineral soil site in Maaninka in eastern Finland cultivated with a perennial crop, (**C**) Low flux period N_2_O emissions (μg N_2_O m^−2^ hr^−1^) fitted as a linear function of the concurrently measured gross ecosystem productivity (mg CO_2_ m^−2^ hr^−1^). The solid line represents the linear least squares fit to the data, values of the slope, intercept, and the coefficient of determination resulting from this fit are shown in the panel.

**Figure 4:**
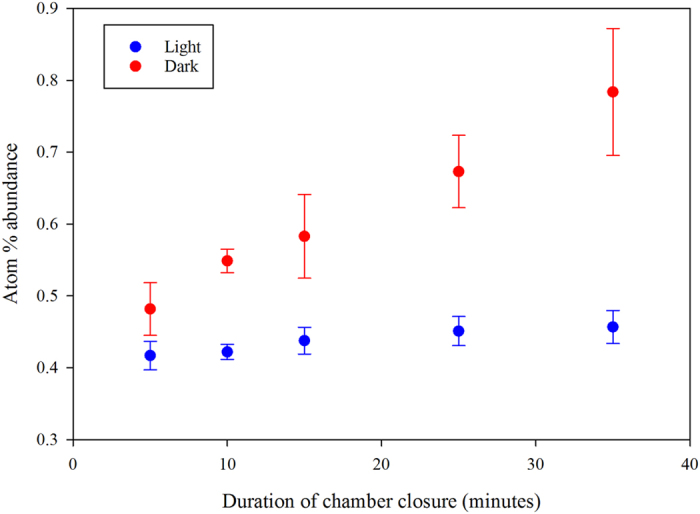
Measurements of atom % abundance following the addition of labelled ^15^N fertilizer to timothy and meadow fescue grass plots located on a mineral site cultivated with a perennial bioenergy crop in eastern Finland. The blue dots represent measurements made with a transparent chamber and the red dots with a dark chamber.
