# Author Correction: Neglecting diurnal variations leads to uncertainties in terrestrial nitrous oxide emissions

**DOI:** 10.1038/s41598-020-63751-9

**Published:** 2020-04-20

**Authors:** Narasinha J. Shurpali, Üllar Rannik, Simo Jokinen, Saara Lind, Christina Biasi, Ivan Mammarella, Olli Peltola, Mari Pihlatie, Niina Hyvönen, Mari Räty, Sami Haapanala, Mark Zahniser, Perttu Virkajärvi, Timo Vesala, Pertti J. Martikainen

**Affiliations:** 1 Department of Environmental and Biological Sciences, Biogeochemistry research group, University of Eastern Finland, Yliopistoranta 1D E, PO Box 1627, Kuopio campus, FI-70211 Finland; 2 Department of Physics, P.O. Box 48, University of Helsinki, Helsinki, 00014 Finland; 3 Department of Food and Environmental Sciences, Division of Microbiology and Biotechnology, P.O. Box 56, University of Helsinki, Helsinki, FI-00014 Finland; 4 Natural Resources Institute Finland, Green technology, Halolantie 31A, Maaninka, FI-71750 Finland; 5 Aerodyne Research, Inc., 45 Manning Road, Billerica, MA 01821-3976 USA; 6 Viikki Plant Science Centre, University of Helsinki, P.O. Box 27, Helsinki, FI-00014 Finland; 7 Department of Forest Sciences, P. O. Box 27, University of Helsinki, Helsinki, 00014 Finland

Correction to: *Scientific Reports* 10.1038/srep25739, published online 09 May 2016

This Article contains an error in Figure 2, where the units of the y-axis ‘mg m^-2^ hr^-1^’ should read ‘µg m^-2^ hr^-1^’. The correct Figure 2 appears below as Figure 1.

**Figure 1:**
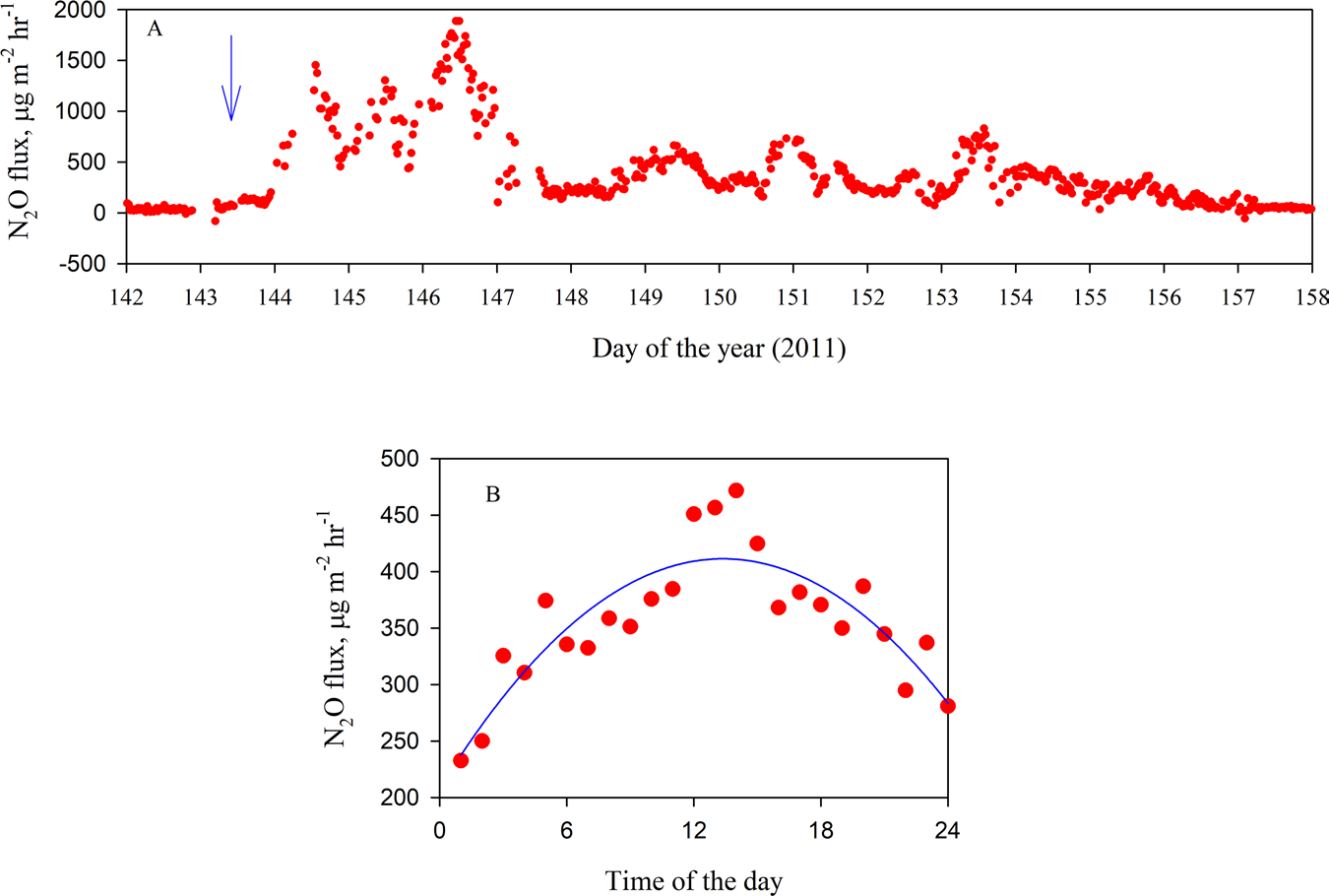
.

