# Supplementary material for: Neglecting diurnal variations leads to uncertainties in terrestrial nitrous oxide emissions
Source: Sci Rep. 2016 May 9;6:25739. doi: 10.1038/srep25739 (PMC4860568; doi:10.1038/srep25739)
Supplement: Supplementary Information [file srep25739-s1.pdf]

Neglecting diurnal variations leads to uncertainties in terrestrial nitrous oxide emissions

Narasinha J. Shurpali, Üllar Rannik, Simo Jokinen, Saara Lind, Christina Biasi, Ivan Mammarella, Olli Peltola, Mari Pihlatie, Niina Hyvönen, Mari Räty, Sami Haapanala, Mark Zahniser, Perttu Virkajärvi, Timo Vesala and Pertti J. Martikainen

Supplementary Information

Soil Physical and chemical characteristics of the study site

The soil type varied between loam and clay loam (clay - 22-34%, silt - 46-64%, sand - 14-30%). According to the World Reference Base for Soil Resources (WRB) system[55], the soil is classified as Haplic Cambisol/Regosol (Hypereutric, Siltic). The soil pH varied from 5.4 to 6.1 within the ploughing depth from the surface to about 30 cm below, electrical conductivity between 960 to 3060 $\mu S\ cm^{-1}$ and soil organic matter content between 3 and 11%. The average C/N ratio in the ploughing depth is 14.9 (ranging from 14.1 to 15.7). The soil particle density is about 2.65 $g\ cm^{-3}$ (0-20 cm soil depth).

Eddy covariance set up

Measurements were conducted by the University of Helsinki (UH) and by the University of Eastern Finland (UEF)[18], operating separate EC systems. The UH measurement setup included a 3-D ultrasonic anemometer (USA-1, METEK GmbH, Elmshorn, Germany) to acquire the wind components. The anemometer was installed on top of a pole, the

measurement height being 2.2 m. The measurement height was raised to 2.4 m on 30.6.2011 due to RCG growth. Gas analysers were situated in an air conditioned cabin located about 15 m east from the anemometer pole. This wind direction (50-110° sector) was, therefore discarded from analysis due to possible disturbances to flux measurements. Sample inlets for gas analysers were located 10 cm below the anemometer. The N_2O instruments operated by the UH were the instrument based on tuneable diode laser (**TGA** - model TGA100A, Campbell Scientific Inc.), and two instruments based on continuous wave quantum cascade lasers, (**ARC** - model CW-TILDAS-CS, Aerodyne Research Inc.[56,57]) see e.g. and (**LGR** - model N_2O/CO-23d, Los Gatos Research Inc.[58]). Sampling lines of ARC and LGR were heated slightly above ambient temperature in order to avoid water from condensing to the lines. TGA had a dryer just before the instrument and no sampling line heating was used.

The UEF set up included a pulsed quantum cascade laser spectrometer (**ARP** – Model QC-TILDAS-76-CS[18], Aerodyne Research Inc., Billerica, MS, USA), an infrared gas analyser (IRGA, Model Li-6262) and a 3-D sonic anemometer (Model R3-50, Gill Instruments, Ltd., Hampshire, UK) for fast response gas concentration and wind component measurements. The heated intake tubes for the laser spectrometer and IRGA were installed on either sides of the sonic anemometer, all mounted on a boom on an adjustable instrument mast. The mast height was set at 2.0 m above the soil surface in the beginning of the campaign. To allow for the increasing plant height, the mast was raised to 2.5 m during mid- June. ARP was set up to measure simultaneously the N_2O, CO_2 and water vapour mixing ratios, while the IRGA was used to monitor CO_2 and water vapour mixing ratios. Both trace gas analysers were calibrated against standard gases minimum once a month during the campaign, in particular ARP was calibrated every 2-3 weeks during summer with two standard gases 299 and 342 ppm.

Eddy covariance data processing and quality control

Measurements were sampled at 10 Hz frequency. Filtering to eliminate spikes was performed according to standard approach[59], where high frequency eddy covariance data were despiked by comparing two adjacent measurements. The spectroscopic correction due to water vapour impact on the absorption line shape was applied along with Webb-Pearman-Leuning (WPL) dilution correction due to water vapour on high-frequency raw concentration output. The correction was not necessary for TGA as a dryer installed after the air intake point on the sampling line dried the air sample before the optical cell. LGR corrected for the water vapour effect by a built-in module in the LGR data acquisition software. Prior to calculating the turbulent fluxes, a 2-D rotation (mean lateral and vertical wind equal to zero) of sonic anemometer wind components was done[60] and all variables were linearly detrended. The EC fluxes were calculated as 30 min co-variances between the scalars and vertical wind velocity following commonly accepted procedures[61]. Then, humidity effect on temperature flux was also taken into account[62]. All data processing was performed with the EddyUH post-processing software (http://www.atm.helsinki.fi/Eddy_Covariance/EddyUHsoftware.php). Prior to analysis data quality screening was performed. In addition to discarding data from the 50-110° wind sector, data quality control procedures included applying the following statistics and selection thresholds: data with N_2O concentration skewness outside (-2,2), or kurtosis outside the (1, 8) range, or Haar mean and Haar variance exceeding 3 were rejected.. Data screening according to night-time turbulence intensity (typically performed using friction velocity threshold) was not done because CO_2 exchange did not exhibit sensitivity to turbulence level, allowing us to assume that vertical turbulent fluxes, corrected for storage change, represent well the ecosystem exchange under all conditions at a given site.

Soil profile measurements of temperature, moisture, oxygen concentration

We installed sensors to measure soil temperature (107-L temperature probe, Campbell Scientific, Inc.), soil moisture (CS-616 water content reflectometer, Campbell Scientific, Inc.) and oxygen concentration (GS Oxygen Sensor KE-25, Figaro USA Inc.) from 5, 10, 20, 30, 45, 60, 80, and 100 cm depths in soil profile. Data were recorded at half-hourly time interval with a datalogger (CR800, Campbell Scientific, Inc.). This measurement system was installed at three different locations in the RCG field in the summer of 2010. Large pits were dug in the field prior to installation. Various sensors were inserted at appropriate depths horizontally along the walls of the pits which were then covered with the soil in the order the soil was removed from the field while digging. It has to be noted that the due to the relatively large size of the collectors, the oxygen sensor measures primarily the bulk soil oxygen content. Thus, the actual oxygen content of soil microsites may not be well represented. This may overestimate the bioavailability of oxygen in the soil. These sensors, nevertheless, are useful in elucidating the spatial and temporal trends in soil aeration.

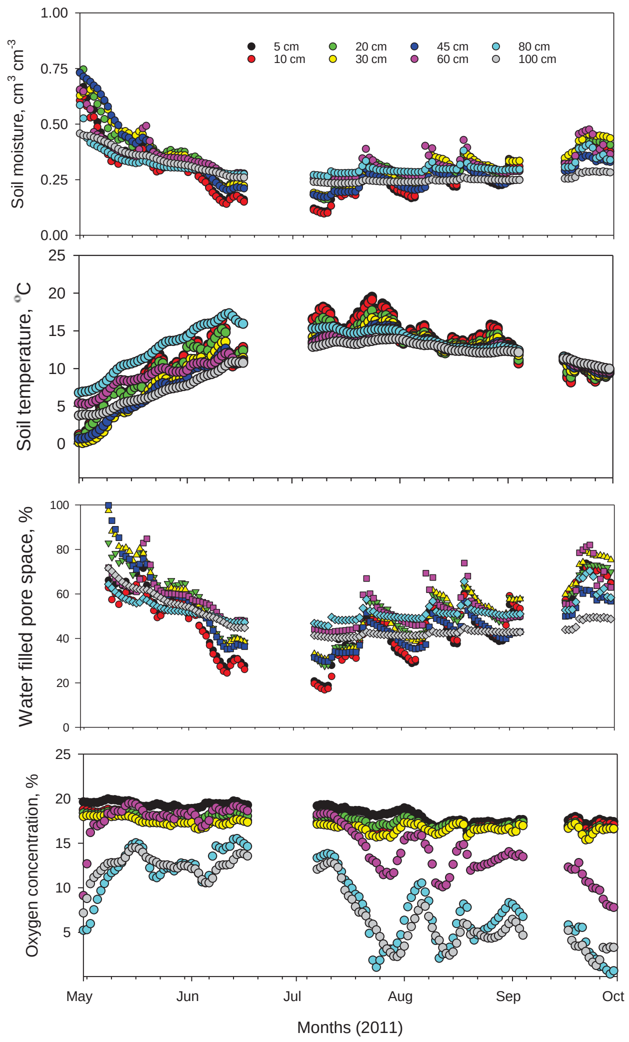

Supplementary Figure S1: Seasonal variations in soil moisture, temperature, water filled pore space and oxygen concentrations in eight different layers of the soil profile (5, 10, 20, 30, 45, 60, 80 and 100 cm). Note that the soil temperatures at 45 cm are not included owing to sensor failure at this depth.

Estimation of water filled pore space (WFPS)

Water filled pore space was estimated using the following formula;

$$WFPS = [TP - \theta_v]$$

Where WFPS is the water filled pore space in %, TP is the total porosity defined as the ratio of average soil bulk density (1.1 g cm^{-3}) and particle density (2.65 g cm^{-3}), and θ_v is the layer wise volumetric soil moisture content (in cm^3 cm^{-3})

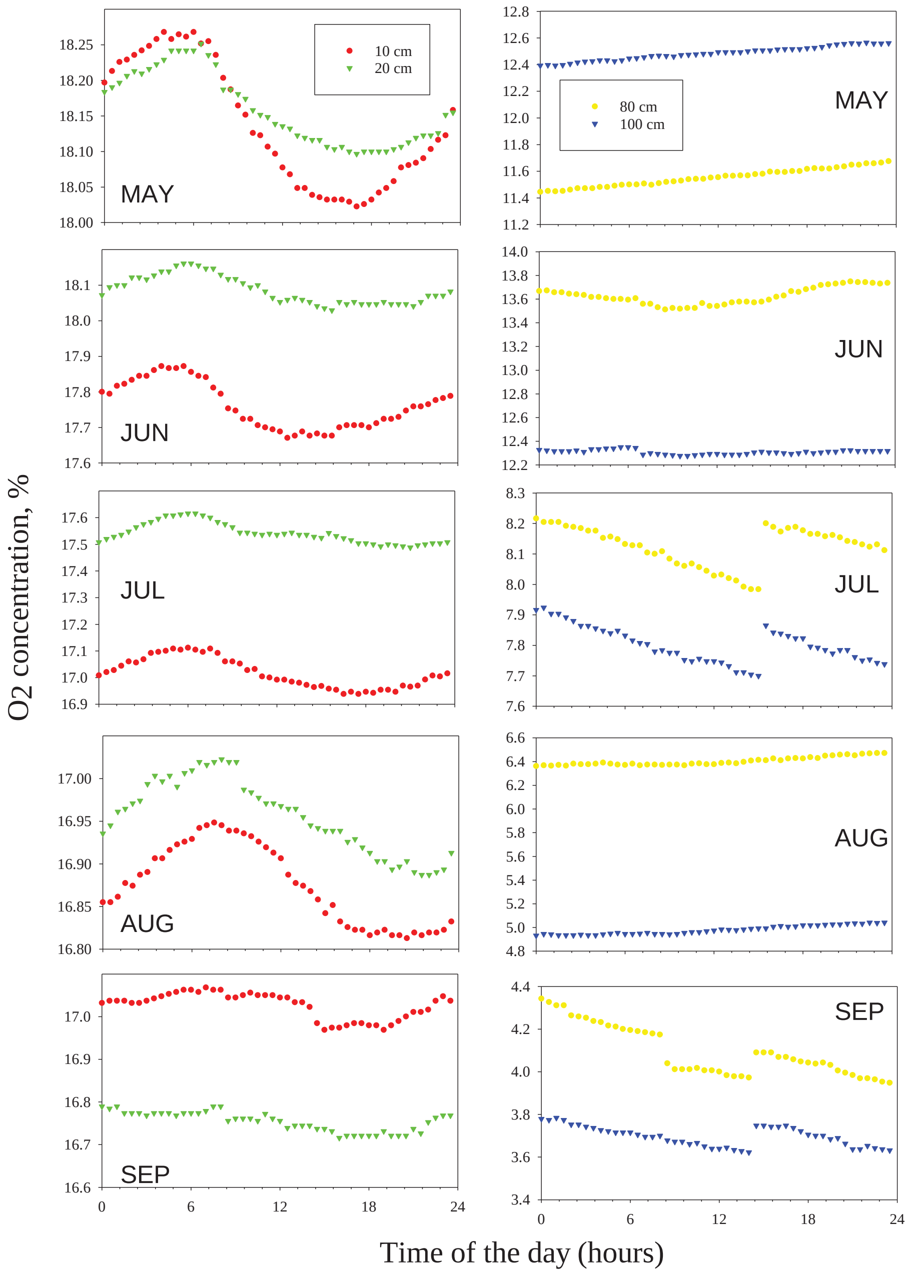

Supplementary Figure 2: Mean monthly diurnal patterns in soil oxygen concentrations at 10 (red), 20 (green), 80 (yellow) and 100 cm (blue).

Analysis of soil NO_3^- content in the field

Soil samples were collected eight times from three replicate locations at 0–15 cm and 15–30 cm depths during the period from April to September 2011 with a soil corer. Soil nitrate (NO_3^-) concentrations were analyzed by an ion chromatograph (DX 120, Dionex corporation, USA) from the soil extractions made with distilled water (25 g of soil fresh weight and 50 of ml distilled water)[63]. A seasonal distribution of measured soil NO_3^- concentrations are shown in Supplementary Figure S1.

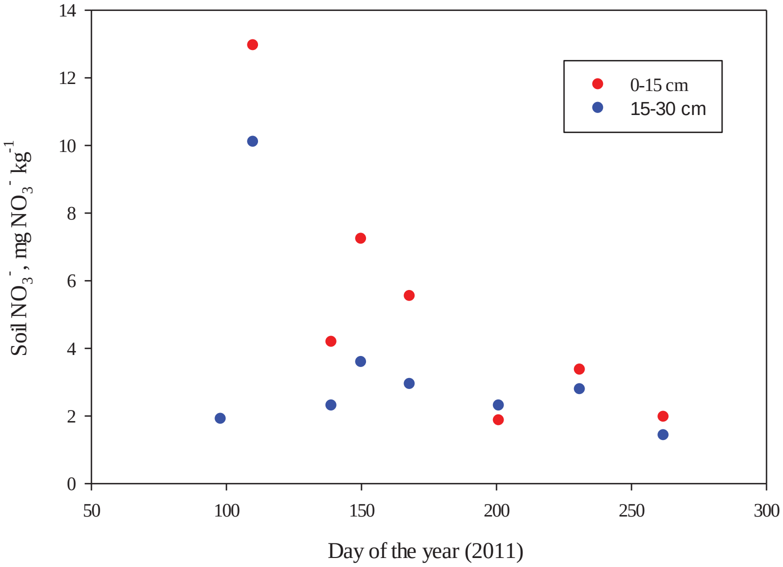

Supplementary Figure S2: Seasonal pattern of soil NO_3^- content at the study site cultivated with a perennial bioenergy crop. These measurements were made at two different depths (0-15 and 15-30 cm) during the 2011 growing season.

Competing Financial Interests statement

The authors declare no competing financial interests.

References

References 1-54 are available in the main text.

55. IUSS Working Group WRB. World Reference Base for Soil Resources 2006, First update 2007, World Soil Resources reports No. 103. FAO, Rome, (2007).
56. Zahniser, M. S., *et al*. Infrared QC laser applications to field measurements of atmospheric trace gas sources and sinks in environmental research: enhanced capabilities using continuous wave QCLs, *Proc. SPIE*, 7222, DOI: 0.1117/12.815172, (2009).
57. Lee, B. H., *et al*. Simultaneous measurements of atmospheric HONO and NO2 via absorption spectroscopy using tunable mid-infrared continuous-wave quantum cascade lasers, *Appl. Phys. B*, **102**, 417–423, (2011).
58. Provencal, R., *et al*. Cavity-enhanced quantum cascade laser-based instrument for carbon monoxide measurements, *Appl. Optics*, **44**, 6712–6717, (2005).
59. Vickers, D. & Mahrt, L. Quality control and flux sampling problems for tower and aircraft data. *J. Atmos. Ocean. Tech*. **14**, 512–526, (1997).
60. Kaimal, J. C., & Finnigan, J. J. Atmospheric Boundary Layer Flows. Their Structure and Measurement, Oxford University Press, New York, (1994).
61. Aubinet, M., *et al*. Estimates of the annual net carbon and water exchange of European forests: the EUROFLUX methodology, *Advances Ecol, Res*. **30**, 113-175, (2000).
62. Schotanus, P., Nieuwstadt, F.T. M. & De Bruin, H. A. R. Temperature measurement with a sonic anemometer and its application to heat and moisture fluxes; *Bound. Lay. Meteorol.* **26.** 81-93, (1983).
63. Maljanen, M. *et al*. Greenhouse gas balances of managed peatlands in the Nordic countries – present knowledge and gaps. *Biogeosciences*. **7**, 2711–2738, (2010).
